# Electrochemical Sensor Nanoarchitectonics for Sensitive Detection of Uric Acid in Human Whole Blood Based on Screen-Printed Carbon Electrode Equipped with Vertically-Ordered Mesoporous Silica-Nanochannel Film

**DOI:** 10.3390/nano12071157

**Published:** 2022-03-31

**Authors:** Kai Ma, Luoxing Yang, Jun Liu, Jiyang Liu

**Affiliations:** 1 Urology and Lithotripsy Center, Peking University People’s Hospital, Beijing 100044, China; Suacaca@hotmail.com (K.M.); hmuliujun@163.com (J.L.); 2 Peking University Applied Lithotripsy Institute, Peking University, Beijing 100044, China; 3 Department of Chemistry, Key Laboratory of Surface & Interface Science of Polymer Materials of Zhejiang Province, Zhejiang Sci-Tech University, Hangzhou 310018, China; 17858903877@163.com

**Keywords:** electrochemical sensor, screen-printed carbon electrode, vertically-ordered mesoporous silica-nanochannel film, uric acid, dual signal amplification

## Abstract

Screen-printed carbon electrodes (SPCEs) bear great potential in the detection of biomarker in clinical samples with low sample consumption. However, modification of electrode surfaces to improve the anti-interference ability and sensitivity is highly desirable for direct electroanalysis of whole blood samples. Here, a reliable and miniaturized electrochemical sensor is demonstrated based on SPCE equipped with vertically-ordered mesoporous silica-nanochannel film (VMSF). To achieve stable binding of VMSF and improve the electrocatalytic performance, electrochemically reduced graphene oxide (ErGO) is applied as a conductive adhesion layer, that is in situ reduced from GO nanosheets during fast growth (less than 10 s) of amino groups modified VMSF (NH_2_-VMSF) using electrochemically assisted self-assembly (EASA). In comparison with bare SPCE, NH_2_-VMSF/ErGO/SPCE exhibits decreased oxidation potential of uric acid (UA) by 147 mV owing to significant electrocatalytic ability of ErGO. The dual signal amplification based on electrocatalysis of ErGO and enrichment of nanochannels leads to enhanced peak current by 3.9 times. Thus, the developed NH_2_-VMSF/ErGO/SPCE sensor enables sensitive detection of UA in the range from 0.5 μM to 180 μM with a low limit of detection (LOD, 129 nM, S/N = 3). Owing to good anti-fouling ability and high selectivity of the sensor, direct and rapid detection of UA in human whole blood is realized with very low sample consumption (50 μL).

## 1. Introduction

Numerous studies have shown that many diseases are closely related to abnormal changes in the concentration of biomarkers. As a class of biomarkers, metabolites are the metabolic product of natural life activities in an organism. Reliable and sensitive detection of metabolites plays an important role in the diagnosis and prevention of diseases [1,2,3,4,5]. For instance, uric acid (UA), as the purine metabolic product, is related to many clinical diseases including renal dysfunction, chronic nephritis, renal insufficiency, gout, and cardiovascular disease, etc. [6,7]. Therefore, monitoring of UA in biological fluids especially in whole blood is critical for long-term evaluation of patient therapy. Among traditional analytical methods, there are two main strategies used to detect serum UA levels, including non-enzymatic and enzymatic methods. The former uses phosphotungstic acid to react with UA under alkaline conditions to produce products with characteristic absorption (λ = 660 nm). The latter applies excess uricase oxidase (UOD) to catalyze the decomposition of UA into allantoin, which bears characteristic absorption (λ = 290 nm). However, these optical methods often require complex blood sample pretreatment procedures, including centrifugation, plasma separation, serum extraction, and so forth. Development of rapid, economical, and sensitive detection methods to achieve direct analysis of UA in whole blood are highly desirable.

Electrochemical detection has the advantages of simple instrumentation, fast response, convenient detection, and potential for easy miniaturization or integration [8,9]. The electrode plays an important role in improving the performance of electrochemical sensors. Compared with conventional solid electrodes (e.g., glassy carbon electrode-GCE, Au or Pt electrode), screen-printed electrodes (SPEs) have attracted much attention owing to their low cost, easy batch fabrication, convenient array and flexible design, and possibility of low sample consumption [10,11,12]. Amongst alternatives, screen-printed carbon electrodes (SPCEs) have great potential in the detection of biomarkers in clinical samples owing to the merits of a wide potential window, low background current, high biocompatibility, and excellent chemical stability [13,14,15]. However, modification of electrode surfaces to improve the anti-interference ability and sensitivity are urgently needed for direct analysis of complex samples with high performance. 

Vertically-ordered mesoporous silica-nanochannel film (VMSF) modified electrodes have illustrated excellent anti-fouling ability and allowed direct electroanalysis in complex samples [16,17]. VMSF has a mechanically stable nanostructure with ultrathin thickness (50–200 nm), long-range ordered and ultrasmall pores (usually 2–3 nm), and ultrahigh pore density (up to 3~12 × 10^12^ cm^−2^) [18,19,20]. Moreover, it has a high surface area as one of solid-state nanofilms [21,22]. The open and uniform nanochannel array displays high molecular permeability in addition to outstanding selectivity towards the size and charge of molecules [23,24,25,26,27,28]. On the one hand, VMSF with ultrasmall nanochannels can keep away larger molecules that could passivate the underlying electrode surface, allowing direct detection of complex samples without sample pre-treatment. On the other hand, a high surface area and uniform surface charge enable large adsorption capabilities towards small molecules with opposite charges. For instance, the silanol groups (Si-OH) on VMSF provide a negatively charged surface, which exhibits an effective response to the cationic analyte while rejecting anions. On the contrary, amino modified VMSF (NH_2_-VMSF) displays positive electricity, which shows strong electrostatic adsorption on negatively charged molecules, resulting in significant enrichment. Therefore, VMSF-modified electrodes have great potential in sensitive detection of complex samples. The integration of VMSF with SPCE will open up enormous opportunities for fast, in situ or on-line detection. However, VMSF exhibits good adhesion only when grown on oxide-based electrode surfaces (e.g., indium tin oxide), possibly by forming covalent bonds with silica. An additional adhesion layer is needed to ensure strong attachment of VMSF to carbon electrodes. Therefore, it is highly desirable to develop facile strategies to stably equip SPCE with VMSF to fabricate reliable and miniaturized electrochemical sensors for sensitive detection of biomarkers in human whole blood.

In this work, we demonstrate the facile fabrication of a reliable and miniaturized electrochemical sensing platform based on SPCE equipped with VMSF, which enable sensitive detection of biomarker in human whole blood. Electrochemically reduced graphene oxide (ErGO), that was in situ synthesized through electrochemical reduction of GO in the preparation of VMSF using an electrochemically assisted self-assembly (EASA) method, was applied as conductive adhesion to stably integrate SPCE with amine groups modified VMSF (NH_2_-VMSF). As the proof-of-concept demonstration, we employed an NH_2_-VMSF/ErGO/SPCE sensor for the detection of UA in human whole blood. Owing to the electrocatalytical capability of ErGO and electrostatic enrichment from nanochannels, the developed sensor exhibits reduced oxidation potential and increased peak current towards UA. Combining the anti-fouling ability of VMSF, the diluted human whole blood was directly dropped onto the NH_2_-VMSF/ErGO/SPCE sensor to achieve rapid and sensitive detection. Compared with VMSF modified electrodes previously reported, the developed sensor exhibits the advantages of low sample consumption (50 μL), which provides great convenience for real application. This work provides a new strategy for the construction of a portable and disposable electrochemical sensor with an anti-fouling layer and high detection performance.

## 2. Materials and Methods

### 2.1. Chemicals and Materials

Monolayered graphene oxide (GO) aqueous dispersion (1 mg/g) was purchased from Hangzhou GaoxiTech (Hangzhou, China). Tetraethyl orthosilicate (TEOS), hexadecyl trimethyl ammonium bromide (CTAB), potassium ferricyanide (K_3_[Fe(CN)_6_]), tetrapotassium hexacyanoferrate trihydrate (K_4_[Fe(CN)_6_]), sodium phosphate dibasic dodecahydrate (Na_2_HPO_4_•12H_2_O), potassium hydrogen phthalate (KHP), D-(+)glucose (Glu), ascorbic acid (AA), uric acid (UA), 3-hydroxytyramine hydrochloride (DA), bovine whole blood albumin (BSA), hemin, starch soluble, and sodium dodecyl sulfate (SDS) were all purchased from Aladdin Chemistry Co., Ltd. (Shanghai, China). Potassium chloride (KCl), calcium chloride (CaCl_2_) and ethanol (EtOH) were purchased from Hangzhou Gaojing Chemistry Co., Ltd. (Hangzhou, China). Sodium chloride (NaCl) and urea were obtained from Tianjin Yongda Chemical Reagent Co., Ltd. (Tianjin, China). Magnesium chloride (MgCl_2_), sodium dihydrogen phosphate dehydrate (Na_2_H_2_PO_4_•2H_2_O) and 3-aminopropyltriethoxysilane (APTES) were obtained from Macklin (Shanghai, China). Screen-printed carbon electrode (SPCE) with a three-electrode system (DRP-C110-U75) was purchased from Metrohm (Bern, Switzerland). Briefly, carbon electrode was used as working and counter electrodes (4 mm in diameter), and silver as reference electrode. Human whole blood (healthy male) was provided by Center for occupational disease prevention and treatment (Hangzhou, China). All solutions were prepared with ultrapure water (18.2 MΩ cm, Millipore). All reagents used in the experiment were of analytical grade without further treatment.

### 2.2. Measurements and Instrumentations

X-ray photoelectron spectroscopy (XPS) analysis was performed using Mg Ká radiation at 250 W and 14 kV (PHI5300, PE, Waltham, MA, USA). The morphology of NH_2_-VMSF was investigated by transmission electron microscopy (TEM) at an acceleration voltage of 100 kV using an HT7700 microscope (Hitachi, Japan). Before analysis, NH_2_-VMSF was gently scraped from the surface of electrode. After being ultrasonicated in EtOH, the obtained dispersion was dropped onto the copper grids. All electrochemical experiments were carried out on an Autolab electrochemical workstation (PGSTAT302N, Metrohm). The scan rate used in cyclic voltammetry (CV) measurement was 50 mV/s. The cyclic voltametric curves were obtained during the first scan (2 segments). The step and modulation amplitude used for differential pulse voltammetry (DPV) were 0.005 V and 0.025 V, respectively. Error bars in the measurements are calculated by the standard deviations of three measurements.

### 2.3. Preparation of NH_2_-VMSF-Modified SPCE Using ErGO as Adhersive Layer

SPCE was firstly electrochemically polished using continuous cyclic voltammetry scanning from 0.4 V to 1.0 V for 10 cycles in H_2_SO_4_ solution (0.05 M). Then, the electrode was thoroughly washed with ultrapure water and dried with N_2_ stream. Subsequently, GO dispersion (15 μL 0.1 mg/mL) was dropped onto the surface of the working electrode and dried at 60 °C. NH_2_-VMSF was synthesized using APTES and TEOS as the mixed siloxanes in presence of CTAB micelles (SM). Briefly, CTAB (1.585 g) and APTES (0.159 g) were added into the mixture containing ethanol (20 mL) and an equal volume of NaNO_3_ solution (0.1 M, pH = 2.6) under stirring. After adjusting the pH of the solution to 3.0 with HCl (6 M), TEOS (2.891 g) was added, and the solution was stirred at room temperature for 2.5 h to obtain the precursor solution. When SPCE was placed in the precursor solution, a voltage of −2.2 V was applied for 5 s. After being thoroughly rinsed using ultrapure water, the obtained electrode with surfactant SM (SM@NH_2_-VMSF/ErGO/SPCE) was aged at 80 °C for 10 h. Then, SM was removed using 0.1 M HCl/EtOH solution (*v*:*v* = 1:1) to obtain electrode with open nanochannels (NH_2_-VMSF/ErGO/SPCE). 

### 2.4. Electrochemical Detection of UA

HAc-NaAc buffer solution (0.1 M, pH 5) was applied as the buffer for the detection of UA. The electrochemical responses of different concentration of UA were recorded using CV or DPV. For real sample analysis, human whole blood was firstly diluted using the buffer by a factor of 50. Then, 50 μL diluted blood was dropped onto NH_2_-VMSF/ErGO/SPCE for detection. 

## 3. Results and Discussion

### 3.1. Stable Coupling of NH_2_-VMSF with SPCE Using ErGO as Adhersive Layer

**Figure 1:**
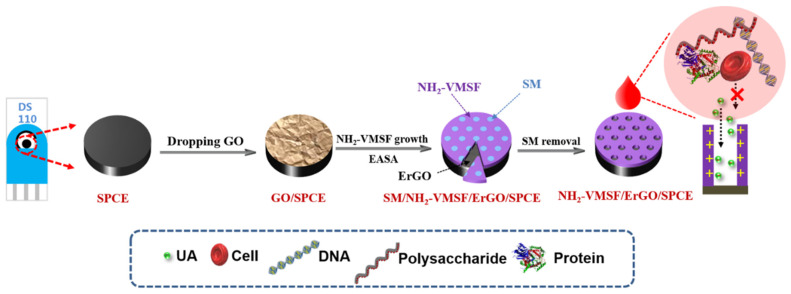
Schematic illustration of the stable coupling of NH_2_-VMSF with SPCE using ErGO as an adhesive layer and direct detection of human whole blood.

As an organic combination of carbon electrode and screen-printed electrode, screen-printed carbon electrodes (SPCEs) have unique characteristics including high chemical stability, wide potential window, good biocompatibility, low cost, easy mass production, and disposable use [13,14,15]. However, it was proven that VMSF cannot be stably grown on carbon electrodes. The introduction of a conductive layer which can create functional oxygenated groups (e.g., -OH groups) on the surface which can form covalent Si–O bonds and multiple hydrogen bonds with VMSF may solve the problem. As illustrated in Figure 1, electrochemically reduced graphene oxide (ErGO) was applied as the conductive and electroactive adhesion layer to realize stable combination of VMSF and SPCE. Electrochemically assisted self-assembly (EASA) method is applied for fast growth of VMSF. Briefly, when SPCE was introduced into the solution containing surfactant micelles (SM) and siloxane precursor, a negative voltage (−2.2 V) was applied to the electrodes. On the one hand, ErGO is formed in situ from the deposited GO nanosheets at the cathodic voltage. On the other hand, protons and water molecules are reduced, resulting in a local increase in pH at the electrode/solution interface, which facilitates the self-assembled growth of silica around the surfactant micelle (SM) template. As the used siloxane precursor contained amino groups, VMSF nanochannels modified with amino groups (NH_2_-VMSF) were obtained. The grown nanochannels were initially filled with surfactant SM (SM@NH_2_-VMSF/ErGO/SPCE). Electrode modified with open nanochannel array can be finally obtained after easy removal of SM using a HCl–ethanol mixture.

**Figure 2:**
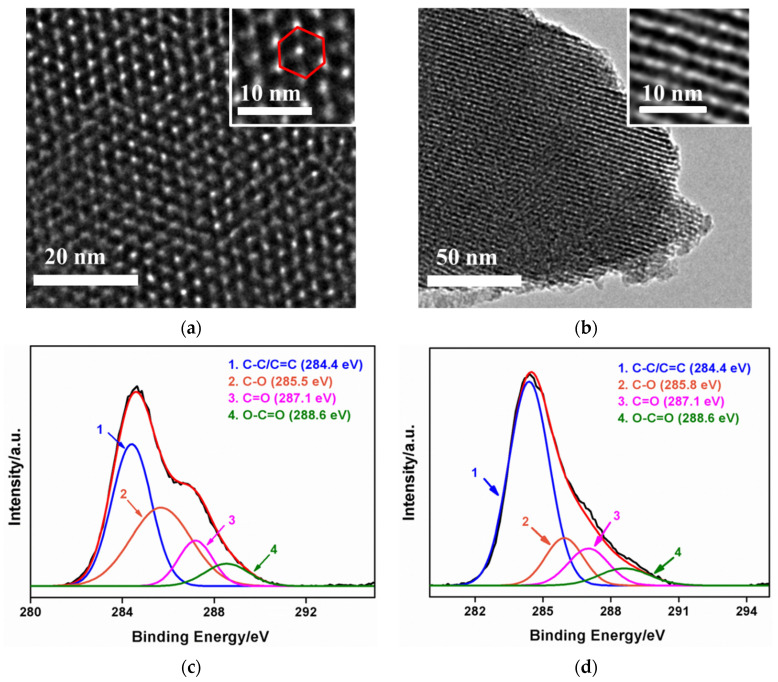
Top-view (**a**) and cross-sectional (**b**) TEM images of NH_2_-VMSF at different magnifications. High-resolution X-ray photoelectron spectrum (XPS) of C1s peaks from GO/SPCE (**c**) and ErGO/SPCE (**d**).

The morphology of NH_2_-VMSF is characterized by transmission electron microscope (TEM). The top view images of NH_2_-VMSF at different magnifications reveal orderly silica nanopores at about 2–3 nm, which are stacked in a hexagonal shape (Figure 2a). In addition, there are no obvious defects in a large area. The pore density is ~7.5×10^12^/cm^2^, which corresponds to a porosity of ~46%. The cross-sectional TEM image of NH_2_-VMSF shows nanochannels parallel to each other (Figure 2b). 

X-ray photoelectron spectroscopy was used to verify the in situ electrochemical reduction of GO while the NH_2_-VMSF grew. Figure 2c,d show high-resolution C1s spectra of GO/SPCE and ErGO/SPCE, respectively. ErGO/SPCE is obtained by dissolving NH_2_-VMSF through treating NH_2_-VMSF/ErGO/SPCE with hot alkali solution. As shown, the C–C/C=C peak obtained after NH_2_-VMSF growth is significantly enhanced, indicating the recovery of sp^2^ carbon structure in graphene. This phenomenon proves that GO on the surface of SPCE is reduced to ErGO during the growth of NH_2_-VMSF. In addition, other peaks corresponding to C–O, C=O, O–C=O groups reveal the existence of oxygen-containing groups on ErGO. Because of the existence of these oxygenated groups, NH_2_-VMSF can be stably grown on an ErGO layer through covalent or hydrogen bonding. At the same time, ErGO can bind with the carbon electrode of SPCE through *π*-*π* interaction and hydrophobic interaction. As known, ErGO has excellent electronic transport properties and intrinsic electrocatalytic activity as a single atom-thick layer of sp^2^ bonded carbon atoms. Thus, ErGO nanosheet can serve as a conductive and electroactive adhesion layer between NH_2_-VMSF and the supporting SPCE.

### 3.2. Enrichment Effect and Electrocatalytic Properties of NH_2_-VMSF/ErGO/SPCE

**Figure 3:**
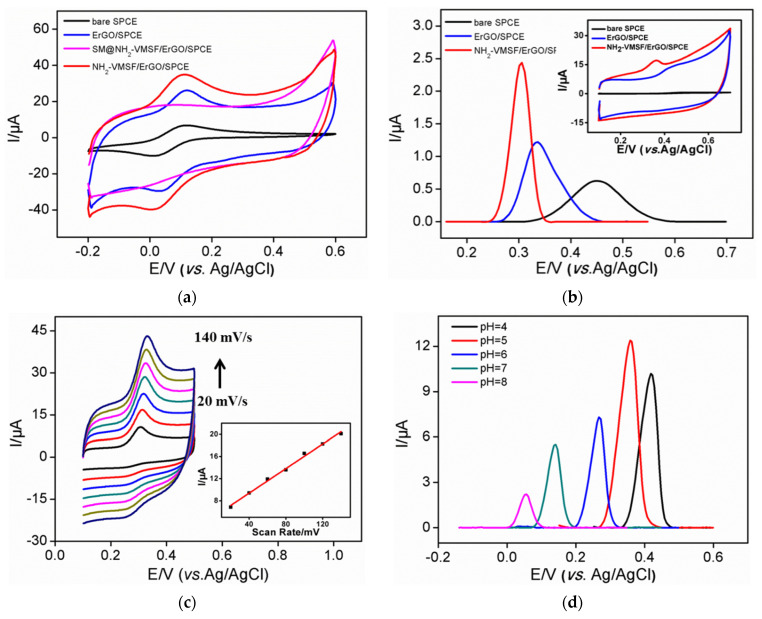
(**a**) Cyclic voltametric curves of Fe(CN)_6_^3−^ on different electrodes. (**b**) Differential pulse voltametric curves of UA (**b**) on different electrodes. Inset is the corresponding cyclic voltametric curve. (**c**) Cyclic voltammetry curves of UA at different scan rate. Inset is the linear regression curve between the oxidation peak current and the scan rate. The increased scan rate between two adjacent lines is 20 mV/s. (**d**) Differential pulse voltametric curves of UA at different pH.

The electrochemical properties of standard electrochemical redox probes (Fe(CN)_6_^3−^) are investigated on different electrodes (Figure 3a). Compared with bare SPCE, increased current was observed on ErGO/SPCE, which was attributed to the promoted electron transfer by ErGO [29,30,31,32]. When SM is filled with nanochannels, no obvious Faraday current signal is measured on SM@NH_2_-VMSF/ErGO/SPCE, indicating the inhibited entry of redox probe to the underlying electrode. This also proves that NH_2_-VMSF completely covers the electrode surface without cracks. When surfactant micelles are removed, NH_2_-VMSF/ErGO/SPCE with open nanochannels exhibit the highest peak current, indicating significant enrichment. This phenomenon is attributed to the electrostatic attraction of NH_2_-VMSF nanochannels. The protonated amino groups act as positively charged sites to realize electrostatic adsorption of negatively charged Fe(CN)_6_^3−^, leading to a significantly enhanced electrochemical signal. 

The electrochemical characterization of UA on different modified electrodes was also investigated by CV and DPV methods (Figure 3b). Compared with that of bare SPCE, the peak potential of UA detected by ErGO/SPCE decreased by 117 mV, suggesting significant electrocatalytic activity of ErGO. At the same time, the peak current increased 1.9 times, indicating a preconcentration effect of ErGO resulting from the π-π interaction between ErGO and UA. In the case of NH_2_-VMSF/ErGO/SPCE, the peak potential of UA decreased by 147 mV and the peak current increased 3.9 times. The good performance of NH_2_-VMSF/ErGO/SPCE are attributed to the high electrochemical activity of ErGO and the enrichment from nanochannels. NH_2_-VMSF with positive charges also exhibit remarkable electrostatic attraction towards negatively charged UA (p*K*_a_ = 3.89). Thus, NH_2_-VMSF/ErGO/SPCE sensors show great potential in sensitive detection of UA with low oxidation potential. 

### 3.3. Optimization of Conditions for the Detection of Uric Acid

Figure 3c shows the cyclic voltametric curves of UA obtained on NH_2_-VMSF/ErGO/SPCE at different scan rates. As shown, the oxidation peak of UA gradually increases with an increase of the scan rate. However, the change of scan rate does not change the electrochemical oxidation potential of UA. This may be attributed to high electronic transport of ErGO and high mass transfer of UA in high-density nanochannels. A good linear relationship is observed between the oxidation peak current and the scan rate (inset in Figure 3c), indicating that the electrochemical reaction process of UA is controlled by adsorption. Thus, the effect of adsorption time on the electrochemical signal of UA is investigated. The peak current increases significantly in the first 3 min. Therefore, enrichment for 3 min was selected in the subsequent experiment. In order to obtain the best detection sensitivity, the pH of the electrochemical electrolyte is also optimized. Figure 3d displays the DPV curves of UA on NH_2_-VMSF/ErGO/SPCE at different pH levels. The oxidation peak current firstly increases with the increase of pH value. Then, the highest oxidation peak current appears at pH 5.0. This may be attributed to the combined effect of pH on the charges of amino groups and UA. When pH rises, the positive charge sites of NH_2_-VMSF decrease due to deprotonation of amino groups. However, the increase of pH leads to an increase of the positive charge of UA. Thus, pH 5 is chosen as the optimal pH for further investigation. 

### 3.4. Sensitive Detection of UA Using NH_2_-VMSF/ErGO/SPCE

**Figure 4:**
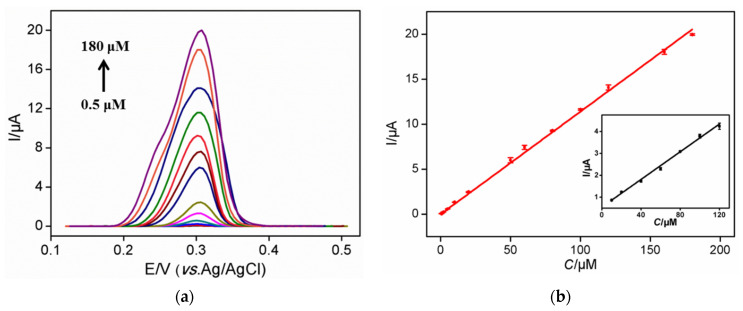
(**a**) Differential pulse voltametric curves of NH_2_-VMSF/ErGO/SPCE to various concentrations of UA (from bottom to up: 0.5; 1; 2; 5; 10; 20; 50; 60; 80; 100; 120; 160; 180 μM) in 0.1 M HAc-NaAc (pH 5). (**b**) The corresponding calibration curves for the detection of UA using NH_2_-VMSF/ErGO/SPCE. Inset is the corresponding calibration curve for the detection of UA using ErGO/SPCE.

NH_2_-VMSF/ErGO/SPCE was used to detect a series of UA solutions with different concentrations. As shown in Figure 4a, the DPV oxidation peak gradually increases with the increase of UA concentration. The oxidation peak current (*I*, μA) is linearly proportional to the concentration of UA (*C*, μM) in the range of 0.5 μM to 180 μM (*I* = 0.114 *C* −0.0137, R^2^ = 0.995) (Figure 4b). The limit of detection (LOD) of 129 nM was obtained at a signal-to-noise ratio of 3. For comparison, linear detection of UA ranges from 10 μM to 120 μM was obtained on ErGO/SPCE (*I* = 0.0315 *C* + 0.546, R^2^ = 0.996) with an LOD of 3.62 μM (S/N = 3). Thus, NH_2_-VMSF/ErGO/SPCE sensor exhibits higher sensitivity and lower LOD, which is mainly due to the further enrichment of nanochannels. The LOD obtained on NH_2_-VMSF/ErGO/SPCE was lower than that obtained on Nafion-modified SPCE [33], nanodiamond modified SPE [34], Uricase/graphene-incorporated chitosan/Prussian blue modified SPCE (Uricase/Chi-Gr/PB/SPCE) [35], uricase-inorganic hybrid nanoflowers-Au nanoparticles modified SPCE (UOx-NFs-AuNPs-SPCE) [36], and Co_3_O_4_-ErGO modified SPE (Co_3_O_4_-ErGO/SPE) [37]. The low LOD of the developed sensor lies in the dual amplification effects resulting from ErGO and nanochannels. 

### 3.5. Anti-Fouling of NH_2_-VMSF/ErGO/SPCE Sensor

**Figure 5:**
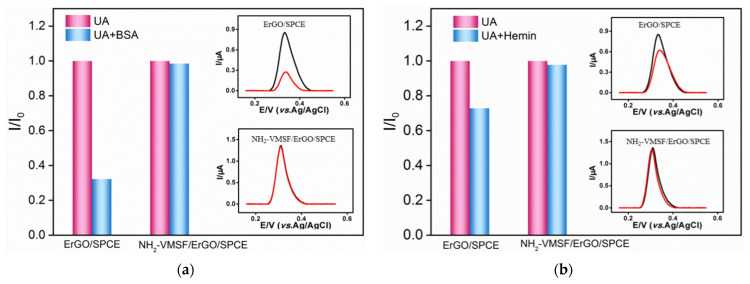

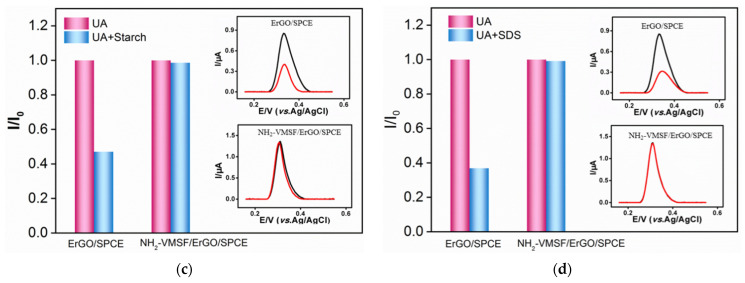
The peak current ratio of UA (10 μM) on ErGO/SPCE or NH_2_-VMSF/ErGO/SPCE in absence (*I*_0_) or presence (*I*) of 50 μg/mL BSA (**a**), hemin (**b**), starch (**c**) or SDS (**d**). Insets are the corresponding differential pulse voltametric curves obtained in single UA (black) or binary mixture (red).

Biological, pharmaceutical and environmental samples usually have complex matrix containing proteins, starch, surfactants, etc., which often contaminate electrode surfaces thus reducing signal stability and accuracy. In addition to using pretreatment (e.g., separation) to remove interfering substances, improving the anti-fouling ability of the electrode is crucial to realize direct electroanalysis of complex samples. Bovine whole blood albumin (BSA), hemin, starch and sodium dodecyl sulfonate (SDS) are applied as model substances to evaluate the anti-fouling ability of the developed sensor. The electrochemical signals of UA in absence or presence of one of these possible interfering substances are given in Figure 5. As shown, the response of UA significantly reduced on ErGO/SPCE when one of the above substances was added, indicating remarkable fouling of the electrode. Whether it is a single uric acid solution or a binary mixture with possible interfering substances, on the contrary, the DPV curves of UA on NH_2_-VMSF/ErGO/SPCE can basically overlap. This phenomenon illustrates the excellent anti-fouling performance of the constructed NH_2_-VMSF/ErGO/SPCE sensor owing to the significant molecular sieving ability of ultrasmall nanochannels.

### 3.6. Stability, Reproducibility, and Selectivity of NH_2_-VMSF/ErGO/SPCE Sensor

**Figure 6:**
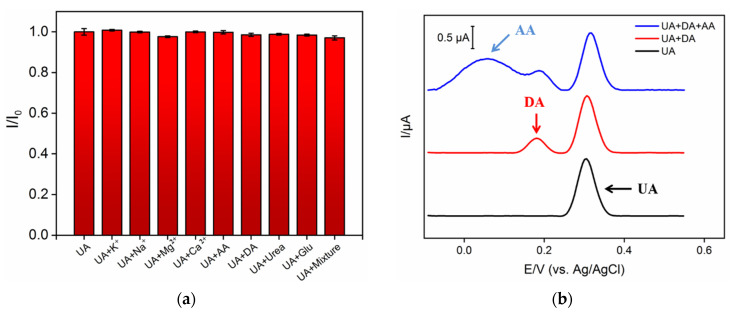
(**a**) The peak current ratio of UA (10 μM) on NH_2_-VMSF/ErGO/SPCE in absence (I_0_) or presence (I) of one or mixture. (**b**) Differential pulse voltametric curves of UA on NH_2_-VMSF/ErGO/SPCE in the absence or presence of AA (10 μM) or DA (10 μM).

The developed NH_2_-VMSF/ErGO/SPCE sensor is remarkably stable, i.e., its electrochemical response to 10 µM UA (*n* = 3) is 97.1% after 7-day storage. For repeatable UA detection (10 µM), the relative standard deviation (RSD) is 2.7% (*n* = 3). The electrode-to-electrode reproducibility was evaluated using five sensors that were independently prepared under the same conditions. An RSD of 0.9% was obtained for the response to 10 µM UA (*n* = 3). In addition to macromolecules, the complex matrix also contains a large number of small molecules. For example, metal ions are widely present in biological and environmental samples. Whole blood or serum samples have high concentrations of glucose (Glu) and urea existing in urine samples. On the other hand, some redox small molecules (e.g., ascorbic acid-AA or dopamine-DA) often co-exist with UA and usually have similar oxidation potentials. To study the selectivity of the sensor, the effects of conventional ions (K^+^, Na^+^, Mg^2+^, Ca^2+^), redox small molecules (AA and DA), and biologically small molecules (Glu and urea) on the electrochemical detection of UA are investigated. As shown in Figure 6a, even when the concentration of possible interfering substances is 20 times higher than that of UA, none of the above single substances or mixtures interfere with the detection of UA. As shown in Figure 6b, the electrochemical peak of UA can be completely separated from those of AA and DA. The high detection selectivity lies in the good potential resolution resulting from the electrocatalytic ability of ErGO. In addition, the charge selectivity of NH_2_-VMSF nanochannels makes it difficult for positively charged substances to reach the underlying electrode.

### 3.7. Detection of UA in Human Whole Blood with Low Sample Consumption

**Figure 7:**
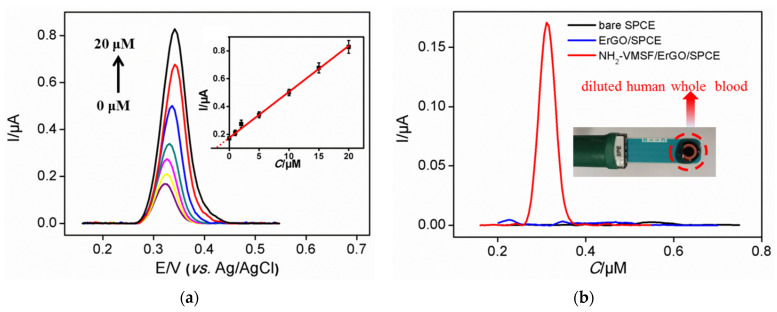
(**a**) Differential pulse voltametric curves obtained on NH_2_-VMSF/ErGO/SPCE in diluted human whole blood with the addition of different concentrations of UA (From bottom to top: 0; 1; 2; 5; 10; 15; 20 μM). Inset is the corresponding calibration curve. (**b**) Differential pulse voltametric curves obtained on NH_2_-VMSF/ErGO/SPCE or ErGO/SPCE in diluted human whole blood. Inset is the digital image of the NH_2_-VMSF/ErGO/SPCE sensor and the detected blood sample.

Owing to easy mass production and low preparation cost, SPCE can be used as a disposable electrode. In addition, its integrated three electrode structure promises great potential in convenient detection with low sample consumption. To verify the feasibility of the developed NH_2_-VMSF/ErGO/SPCE sensor in practical applications, the detection of UA in human whole blood was investigated (Figure 7). For comparison, the detection using ErGO/SPCE was also studied. Human whole blood was directly detected after dilution by a factor of 50. Then, 50 μL of diluted blood sample was directly dropped onto the modified SPCE (inset in Figure 7a). As shown, no signal was observed on ErGO/SPCE because of the significant fouling by the complex matrix of whole blood (Figure 7a). However, a characteristic oxidation peak appears on NH_2_-VMSF/ErGO/SPCE sensor due to the high selectivity and good anti-fouling ability of the electrode (Figure 7a). The concentration of UA in whole blood was calculated by standard addition method (also linear extrapolation method). After adding different concentrations of UA into the diluted whole blood, the electrochemical determination was conducted (Figure 7b). Through the intercept between the linear regression line of oxidation peak current and UA concentration, the concentration of UA in the diluted whole blood was 5.30 μM. Thus, the concentration of UA in undiluted whole blood was concluded to be 265.0 μM. The recovery rate of the other three artificial added UA concentration was between 99.0–107.0% with low relative standard deviation (RSD, ≤2.3%, Table 1), indicating good reliability and accuracy in the direct measurement of UA in real complex samples. In comparison with other electrochemical sensors for UA detection, the developed NH_2_-VMSF/ErGO/SPCE sensor exhibits the advantages of simple preparation, high sensitivity, good anti-fouling ability and low sample consumption.

## 4. Conclusions

In summary, we have developed a reliable and miniaturized electrochemical sensor for sensitive detection of uric acid (UA) in human whole blood based on a screen-printed carbon electrode (SPCE) equipped with a vertically-ordered mesoporous silica-nanochannel film (VMSF). Electrochemically reduced graphene oxide (ErGO) reduced in situ from GO during the growth of VMSF acts as the conductive adhesive layer, offering stable binding of VMSF on SPCE. In addition to the electrocatalysis effect of ErGO, electrostatic enrichment of UA was further realized through amino group modified VMSF nanochannels, that were rapidly synthesized using electrochemically assisted self-assembly (EASA). In comparison with bare SPCE, NH_2_-VMSF/ErGO/SPCE exhibited decreased oxidation potential by 147 mV owing to significant electrocatalytic ability of ErGO. The dual signal amplification based on electrocatalysis of ErGO and enrichment by nanochannels also leads to a significantly enhanced peak current, namely a 3.9-fold increase. Thus, the developed NH_2_-VMSF/ErGO/SPCE sensor enabled sensitive detection of UA with a low limit of detection. Owing to good anti-fouling ability and high selectivity, direct detection of UA in human whole blood was realized with very low sample consumption (50 μL). The established strategy here for facile and efficient fabrication of nanochannel equipped SPCE can provide new methods for direct analysis of complex samples with small sample consumption and high detection performance. 

## Figures and Tables

**Table 1:** Determination of UA in diluted human whole blood samples.

Sample ^a^	Spiked (μM)	Found (μM)	RSD (%)	Recovery (%)
Human whole blood ^a^	0	5.30	1.6	
	1.00	6.37	2.0	107
	5.00	10.3	2.2	100
	10.0	15.2	2.3	99.0

^a^ Samples were diluted 50 times.

## Data Availability

The data presented in this study are available on request from the corresponding author.
